# Supplementary material for: Myosoft: An automated muscle histology analysis tool using machine learning algorithm utilizing FIJI/ImageJ software
Source: PLoS One. 2020 Mar 4;15(3):e0229041. doi: 10.1371/journal.pone.0229041 (PMC7055860; doi:10.1371/journal.pone.0229041)
Supplement: S3 File — (PDF) [file pone.0229041.s003.pdf]

About Myosoft:

Myosoft is an ImageJ-based macro that can be used to analyze muscle fiber size and type automatically. Myosoft accepts up to 4-channel images, identifies muscle fiber boundaries and measures fiber cross-sectional area, and categorizes each fiber according to the channels available. Myosoft is freely available for download here. Myosoft is designed to be user-friendly while also retaining some ability for user input, specifically with regard to morphometric gates that the macro uses to identify objects of interest (e.g. muscle fibers). Therefore, the user will receive several prompts when the macro is run.

Myosoft is built to handle (up to) 4 channel images, with a membrane counterstain on 1 channel, and myosin heavy chain isoforms (or other intracellular proteins) on the remaining 3. By default, Myosoft will prompt the user for the channel number corresponding to the counterstain, type I, type IIa, and type IIb fibers (more detail in section 10). If you are not using all 4 channels, simply provide the numbers for the channels you are using and leave the others blank.

A detailed description of Myosoft outputs is given in the results section below. Briefly, outputs include: (1) Images of each channel with an overlay of "fiber" boundaries (ROIs), (2) ROI.zip files for each single fiber type and mixed fiber type that can be opened in FIJI (3) a .csv spreadsheet (Excel compatible) that includes all measurements taken by Myosoft for each single fiber type and mixed fiber type, (4) a color-coded version of the input image where fibers are pseudo-colored according to fiber cross-sectional area (CSA, a scale is also saved in the results, showing the colors assigned to fibers of various sizes).

If you would like to adapt the Myosoft code for a new application, the macro can be edited in FIJI **Plugins>Macros>Edit>Myosoft.ijm** (select the .ijm file from its source folder). The source code is annotated by the author for clarity.

Requisites for Myosoft

FIJI (Fiji Is Just ImageJ) version 2.0 with Java 1.8 or newer

- The latest version can be downloaded from https://imagej.net/Fiji/Downloads.
- The version can be checked in the Fiji application

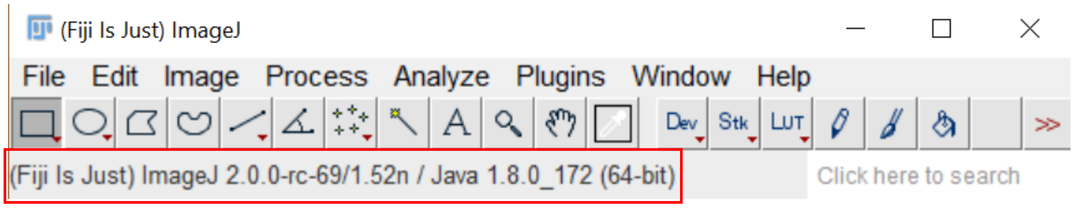

Trainable Weka Segmentation (TWS) v3.2.33

- This should come with the latest FIJI download and can be found in **Plugins>Segmentation> Trainable Weka Segmentation**
- Version of TWS can be found at the top left corner of the window

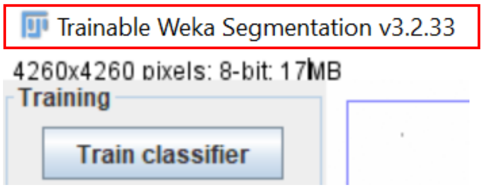

Several FIJI update sites must be added. These include:

BioVoxxel Update

- This will install the Watershed Irregular Features plugin and Biovoxxel Extended Particle Analyzer.

Bar Color Coder

- Note: BAR is a suite of plugins with a variety of functionalities. Once the BAR update site is enabled, “BAR” will appear above the toolbar in the primary FIJI menu.

How to add update sites: (See below images)

- **FIJI > HELP > UPDATE (Not “Update ImageJ”)**
  - Note: when selecting “Update” from the “Help” menu, FIJI will automatically run an updater. The user must wait for the updating to complete (usually a few minutes or less) before the option to “Manage update sites” is displayed.
- **Manage Update Sites:**
  - **Check BioVoxxel** and **check BAR**
  - **Click: Close**
  - **Click: Apply changes**
  - **Restart FIJI**

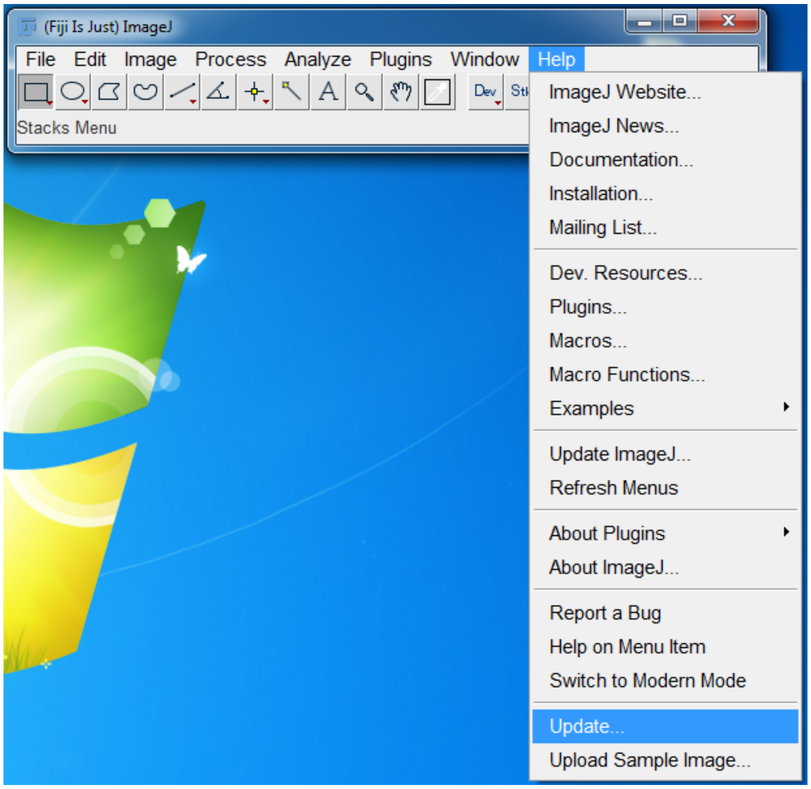
(Fiji Is Just) ImageJ
File
Edit
Image
Process
Analyze
Plugins
Window
Help
Stacks Menu
ImageJ Website...
ImageJ News...
Documentation...
Installation...
Mailing List...
Dev. Resources...
Plugins...
Macros...
Macro Functions...
Examples
Update ImageJ...
Refresh Menus
About Plugins
About ImageJ...
Report a Bug
Help on Menu Item
Switch to Modern Mode
Update...
Upload Sample Image...

- 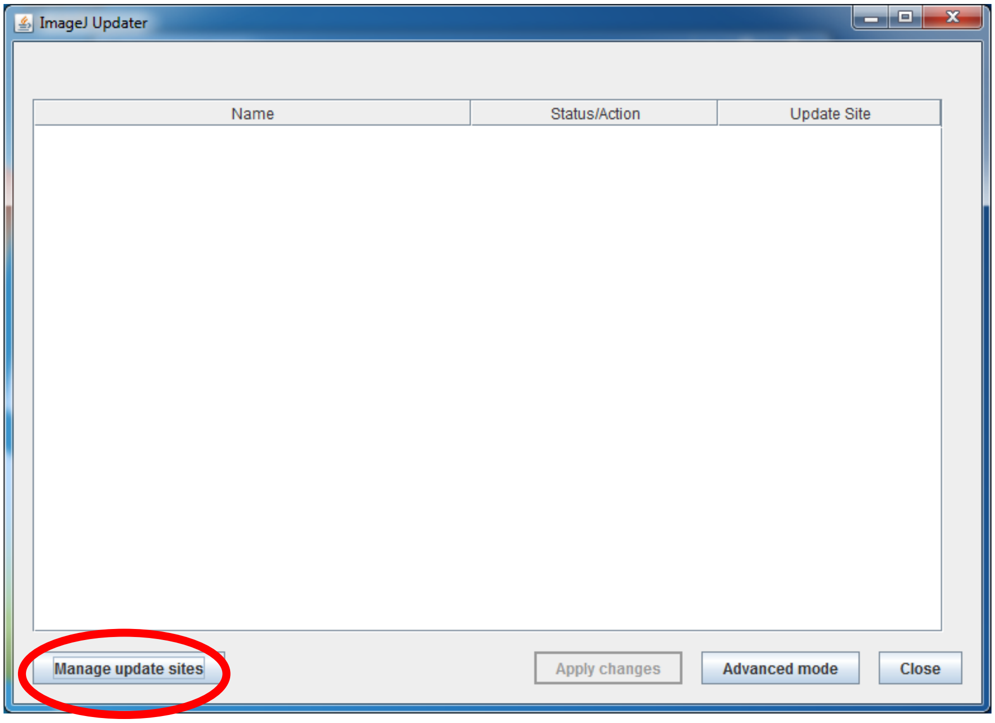
ImageJ Updater
Name
Status/Action
Update Site
Manage update sites
Apply changes
Advanced mode
Close

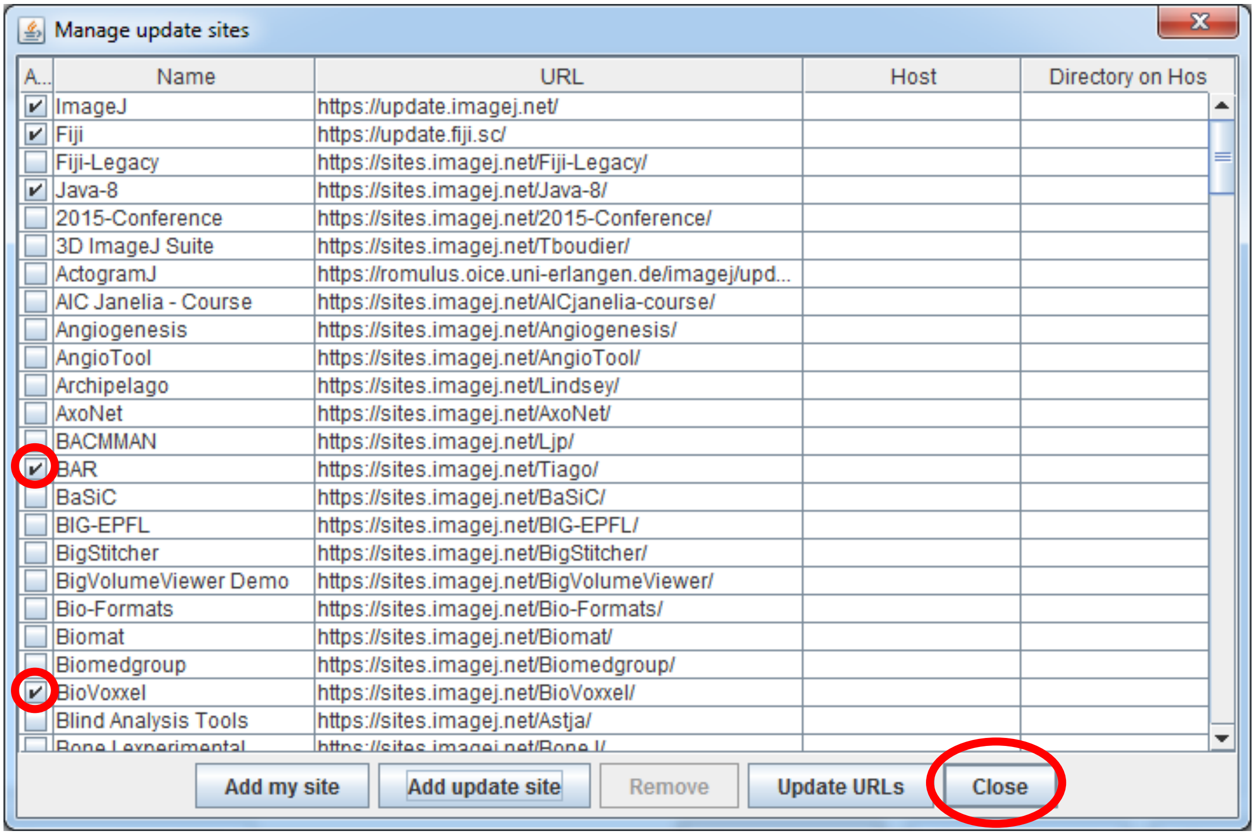
Manage update sites
A...
Name
URL
Host
Directory on Hos
ImageJ
https://update.imagej.net/
Fiji
https://update.fiji.sc/
Fiji-Legacy
https://sites.imagej.net/Fiji-Legacy/
Java-8
https://sites.imagej.net/Java-8/
2015-Conference
https://sites.imagej.net/2015-Conference/
3D ImageJ Suite
https://sites.imagej.net/Tboudier/
ActogramJ
https://romulus.oice.uni-erlangen.de/imagej/upd...
AIC Janelia - Course
https://sites.imagej.net/AICjanelia-course/
Angiogenesis
https://sites.imagej.net/Angiogenesis/
AngioTool
https://sites.imagej.net/AngioTool/
Archipelago
https://sites.imagej.net/Lindsey/
AxoNet
https://sites.imagej.net/AxoNet/
BACMMAN
https://sites.imagej.net/Ljp/
BAR
https://sites.imagej.net/Tiago/
BaSiC
https://sites.imagej.net/BaSiC/
BIG-EPFL
https://sites.imagej.net/BIG-EPFL/
BigStitcher
https://sites.imagej.net/BigStitcher/
BigVolumeViewer Demo
https://sites.imagej.net/BigVolumeViewer/
Bio-Formats
https://sites.imagej.net/Bio-Formats/
Biomat
https://sites.imagej.net/Biomat/
Biomedgroup
https://sites.imagej.net/Biomedgroup/
BioVoxxel
https://sites.imagej.net/BioVoxxel/
Blind Analysis Tools
https://sites.imagej.net/Astja/
Add my site
Add update site
Remove
Update URLs
Close

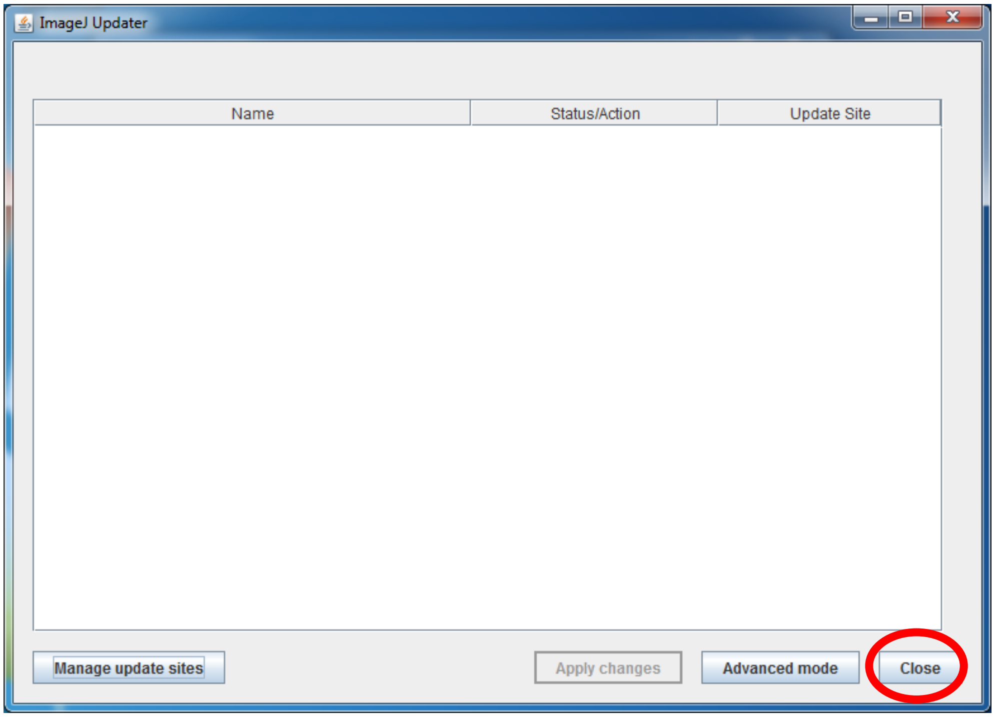
ImageJ Updater
Name
Status/Action
Update Site
Manage update sites
Apply changes
Advanced mode
Close

Using Myosoft – Windows and Mac

- Download and initialization

- Myosoft is freely available for download here or from supplemental files.

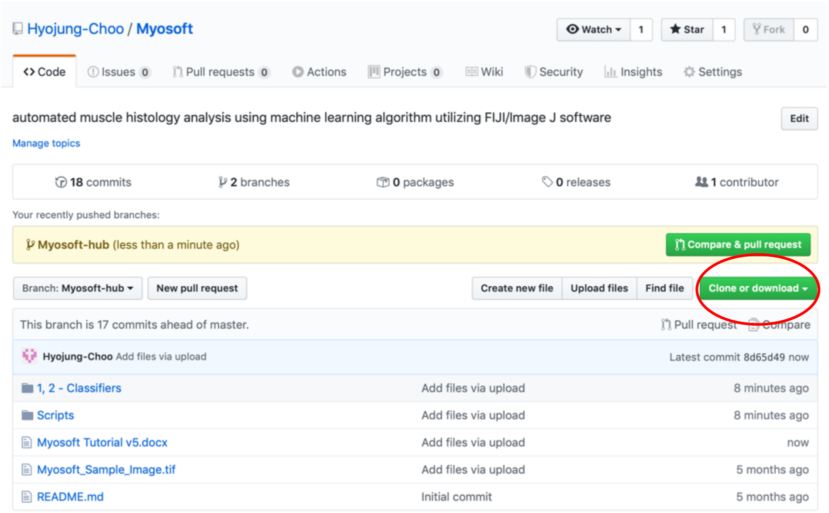

  - After following the link, select "Clone or download"
  - Select Download ZIP
  - After the user locates the ZIP file on their computer, right click and select extract all
- Save the Myosoft Hub folder to an easily accessible location
  - The directory should be (for example) C:\Myosoft Hub for Windows, Document/Myosoft Hub for Mac
  - A number of subfolders are included in the parental "Myosoft Hub" folder. These are explained below.
    - "1,2-Classifiers" – Contains "simple fiber outline classifer.model" and "iterative fiber classifier.model" which are pre-trained segmentation algorithms that Myosoft uses to detect object boundaries (this is the machine-learning component of Myosoft). This folder also contains "iterative classifier centronuceli.model" which is used by the central nuclei counter macro.
    - "Scripts" – Contains (1) "Myosoft.ijm" – the muscle fiber analysis macro, (2) "images to hyperstack.ijm" – The macro which converts single ImageJ images into a hyperstack, and (3) central nuclei counter.ijm – The macro that detects centrally nucleated myofibers.
    - "Myosoft_Sample_Image.tif" – A sample image to run through Myosoft to test its function.

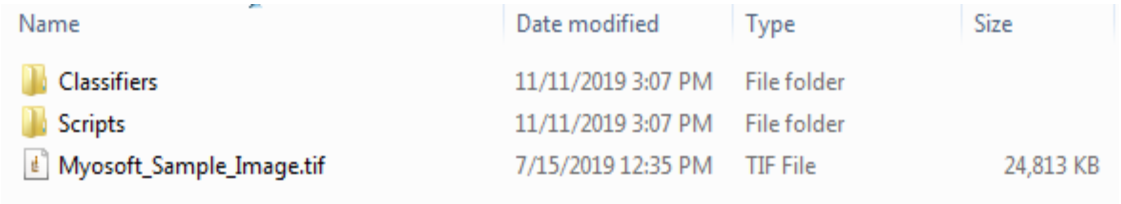

Above: Default Myosoft folders.

How to Use Myosoft

1. **Open a multichannel image in FIJI** (Myosoft_Sample_Image.tif is in the Myosoft Hub folder). When a multi-channel image is opened in FIJI, the bio-formats Import Options window will open. Open your image as a hyperstack, and do not split channels (see red circles below). **It is highly recommended that you know the scale of your image (pixels per micron) before running Myosoft.** You will be asked to supply this value in Step 7.

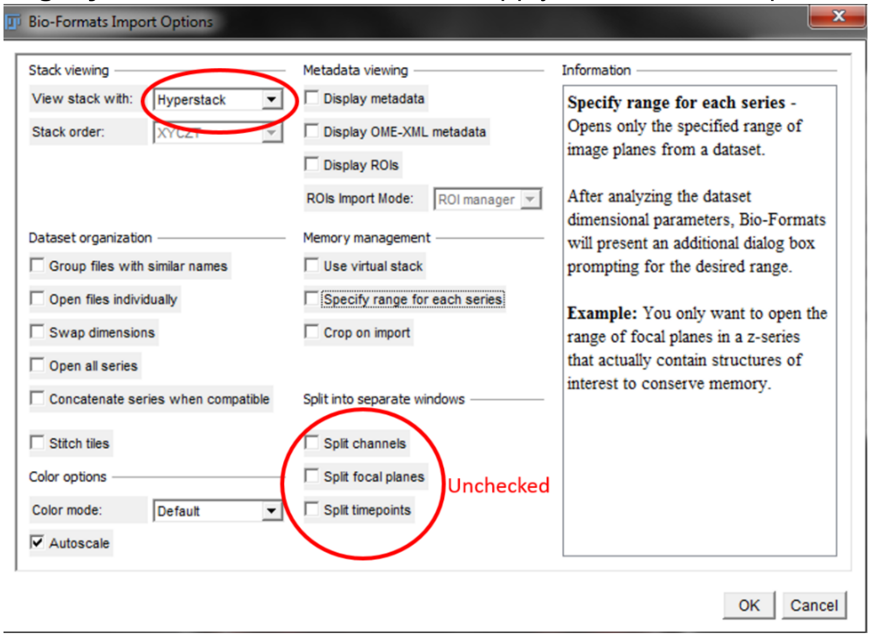

- **If you are starting with single channel images**: We provide a macro "images to hyperstack.ijm" to combine your images and format them as a hyperstack in the Myosoft Hub\scripts folder. Open single channel images. Go to **Plugins>Macros>Run and navigate to hyperstack.ijm in Myosoft Hub\Scripts.** Click Run and save the resulting image before running Myosoft.
- **Image Cleanup**
  It is highly recommended that users clear any large objects that are not cells from their images. This includes nerves, blood vessels and any other large circular objects that are not muscle fibers. This will prevent these objects from being identified as cells during analysis.
  - To do this: Select polygon selection or freehand selection and outline the object to be removed.

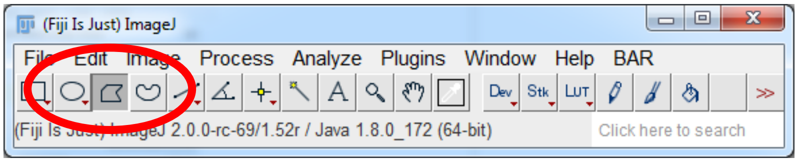

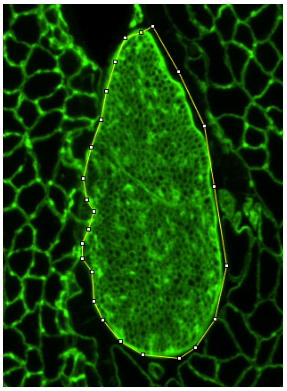

- Select Edit>Clear>Yes.

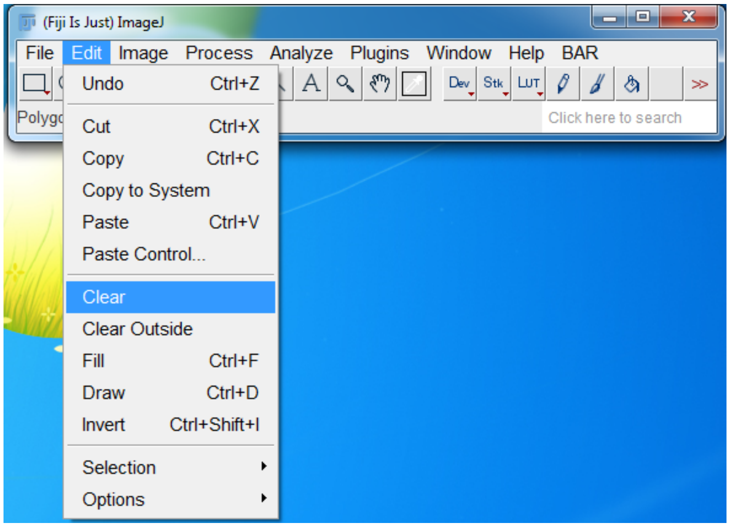

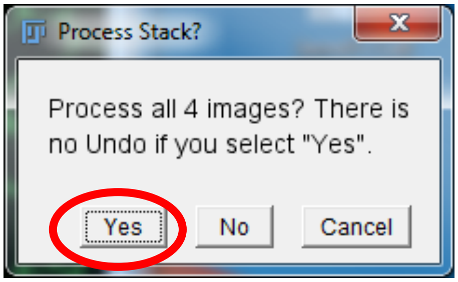

2. **Open the Myosoft.ijm macro** by dragging it onto FIJI or by going to Plugins>Macros>Run and navigate to Myosoft.ijm in Myosoft Hub\Scripts.

- Click Run.

- First, you will receive a prompt regarding the location of the “simple classifier” and the “iterative classifier”. The default location of the classifiers is \Myosoft Hub\Classifiers. You will receive two separate prompts for each classifier. For each prompt, select the \Myosoft Hub\Classifier\ folder and select OK.

- Next, you will receive several prompts regarding where to save output data. Make and name folders for each image that will be analyzed. When Myosoft is run, you will be prompted to choose a directory to save different data. Prompts are:

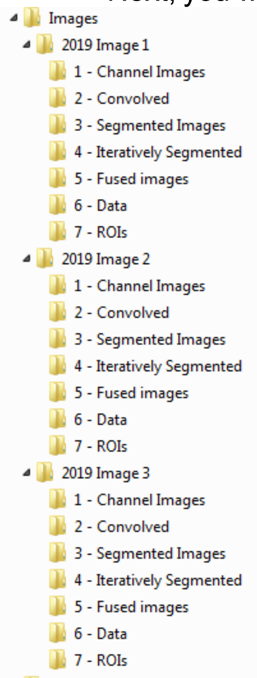

- “choose place to save single channel images” Create subfolder in \”insert image name”\1 - Channel Images.
- “choose place to save convolved images” – Create subfolder in \”insert image name”\2 - Convolved
- “choose place to save segmented images” – Create subfolder in \”insert image name”\3 - Segmented Images
- “choose place to save iteratively segmented images” – Create subfolder in \”insert image name”\4 - Iteratively Segmented Images
- “choose place to save fused images” – Create subfolder in \”insert image name”\5 - Fused Images
- “choose place to save fiber type and morphometry data” – Create subfolder in \”insert image name”\6 - Data
- “chose place to save ROIs” – Create subfolder in \”insert image name”\7 - ROIs

Left: Example subdirectories for multiple samples.

Tip: We recommend to make empty subfolders as template folders and copy and paste under "insert image name (which is made by user)" folder.

3. **Add/change values of morphometric gates.**
Myosoft uses the Extended Particle Analyzer in the BioVoxxel Toolbox to set several "gates," or exclusion criteria for objects that are classified as myofibers but are probably not myofibers or *are* myofibers, but in an improper orientation. For example, the minimum area gate would exclude small blood vessels or nerves, while the maximum area and circularity gates would exclude oblique or longitudinal myofibers within the section.

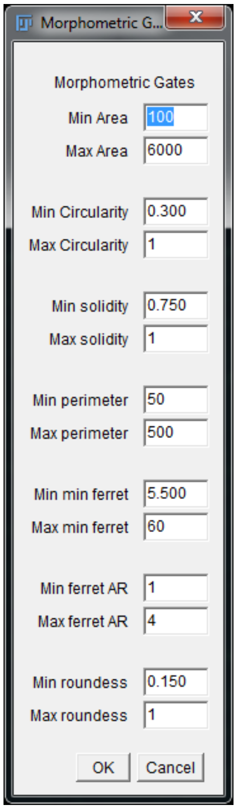

Above: Default morphometric gate values

a. Min/Max area – default range is 100-6000μm^2. Ideally, this range should be tested on a control section, and small and large values should be examined to ensure that these gates are appropriate for your sample. As a general rule, fibers of ~5000μm^2 are quite large and appear at the upper end of ranges reported in the literature. (Perimeter)^2
b. Min/Max circularity – Circularity = $\frac{4\pi(Area)}{(Perimeter)^2}$; a two-dimensional area:surface measurement. For a perfect circle, this value would be 1.
c. Min/Max solidity – Solidity = $\frac{Area}{Convex\ Area}$; in other words, what is the ratio of the true area of your object to the area it would have if it were imagined as a convex polygon (concavities extended outward)?
d. Min/Max perimeter – measures the perimeter of your object.
e. Min/max minimum feret distance – minimum feret distance is the shortest possible distance between any two tangent lines of your object perimeter. For an ovoid shape, this would be the length of the short axis.
f. Min/max feret AR – the aspect ratio (AR) of ferets for your object, that is, $\frac{FeretMax}{FeretMin}$. For an ovoid shape, this would be the ratio of the axes.
g. Min/max roundness – Roundness = $\frac{4Area}{\pi(\max diameter)^2}$; this formula compares how closely your area matches the area of a perfect circle (value for perfect circle is 1).

The Extended Particle Analyzer allows for more gates than those included as defaults in the Myosoft code. To view these, and to see some pictoral representations of the gates used, check here.

4. **A prompt will appear asking for an ROI expansion factor.** See Fig. 1, Step 8 in the paper to see an example of 4 pixel ROI expansion. Any integer value (positive or negative) is acceptable (negative values will shrink ROIs). We recommend starting with a value of 4, which is the default, and adjusting only if this does not suit your needs.

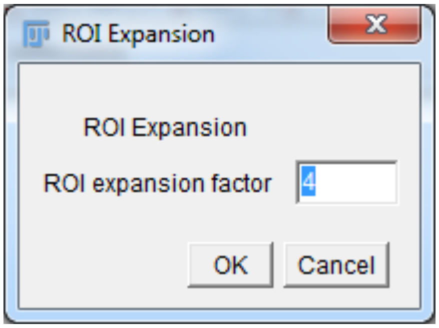

Why is this done? The segmented images show what the classifier views as the probability that a given pixel in the image represents the boundary of an object (black p=1, white p=0, gray values in between). An auto-threshold algorithm is applied to this probability map to generate a binary image that can be analyzed with the extended particle analyzer. Typically, the classifiers will view several pixels at the cell boundary as

plausibly marking the boundary, which is why the iteratively segmented images appear to have thick dark borders around the fibers. ROI expansion moves the ROI outward in all directions by the number of pixels chosen with this prompt to account for excess negative space.

5. **A prompt will appear asking you for the scale of your image.**
IMPORTANT: It is highly recommended that you know the scale (pixels per micron) of your image before running Myosoft. If you do not supply the correct scale, then CSA values generated in Myosoft will not be in real spatial units. As a default, a value of 1.100px/micron is provided (**this is the scale value of the sample image provided in \Myosoft Hub**), but it is likely that your image is scaled differently.

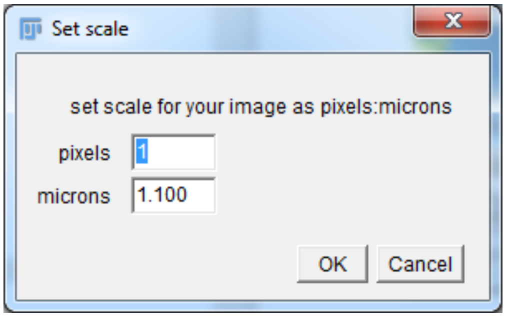

6. **A prompt will appear asking you for Color Coder parameters.**
Once Myosoft has completed fiber CSA analysis, it will automatically run an ROI Color Coder plugin (from BAR suite of plugins). This will provide a color-coded mask of your input image where fibers are assigned colors based on their CSA. This is an aid for visual inspection of your sample's fiber CSA distribution, which is meant to complement a graphical representation of CSA values.

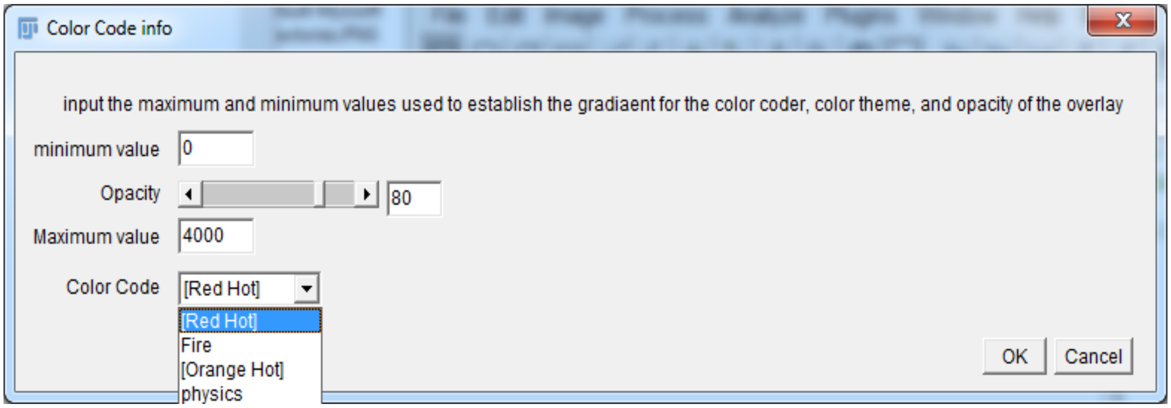

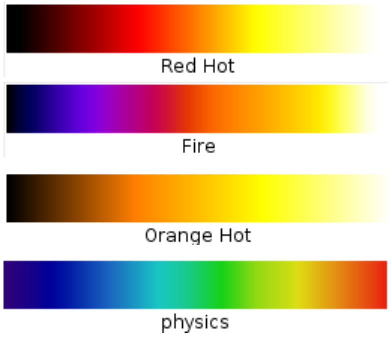

Small -----------------------> Large
Above: Color code LUT options.

a. Minimum value – the lowest area value to which you want to assign a unique color. All objects below this value will receive the same color as the minimum.
b. Opacity – opacity value for colorization. The color-coded ROIs are overlaid on your original image (but saved under a different name), so if you wish to be able to see the original image objects, use a lower opacity value (that is, less than 50). Normally, this is unnecessary, and a value of 80-100 will nicely mask your image.
c. Maximum Value – the highest area value to which you want to assign a unique color. All objects larger than this value will receive the same color as the maximum. Default is 4000
d. Color code – this is the LUT that will be applied to the data. The options are: Red Hot, Fire, Orange Hot, and physics. A default range of 0-4000 is set for fiber sizes.

This means that fibers of ~no size will appear black, while fibers 4000um or greater will appear white. You may wish to adjust this range to better fit your own data.

- The color-coded mask is saved in the location you chose for data (step 2 above) as "InputImageNameHere_referenceImg_ColorCode".

7. **A prompt will appear asking for the position (within the hyperstack) of the membrane counter stain, the type IIa, type I, and type IIb channels.**
1 corresponds to the left-most position, and 4 corresponds to the right-most position. You can check the position using cursor keys or the scroll bar at the bottom of hyperstack image window. If you are not using all 4 channels, provide the numbers for the channels you are using and leave the others blank.
***Note:** Myosoft will still "analyze/provide results" for blank channels, but, it just duplicates analysis of channel 1, and the data for these duplicates can be ignored. The channel names are only names. They do not affect the analysis in any way, but the results will be stored in .csv files that bear the names of the channels. If you are not staining type I, type IIa, and type IIb fibers, we would recommend running Myosoft with these default labels and simply keeping track of which of those labels corresponds to each of your real stains. It is possible to change the names within the code, but that process is slightly complicated.

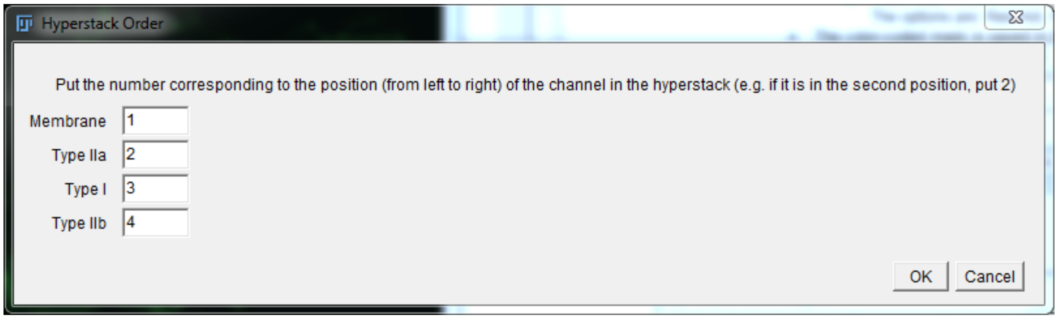

8. **The input image will next be cut into 16(4x4) sub-images by default.**
This step is included to guarantee that your machine has the necessary computing power for Myosoft to operate. Running large images through the classifier is resource intensive and inefficient. Instead, Myosoft will run 16 smaller images through the classifier consecutively. A prompt will appear asking how many divisions you would like to make of your input image.
The default of 4 will cut the input image into 16 (4x4) sub images. However, Myosoft will accept any value from 1 to 5 (resulting in 1-25 sub-images). Choose higher numbers (4 or 5) if the starting image is large and your computer has ≥8GB RAM. If the starting image is small, fewer divisions are necessary.

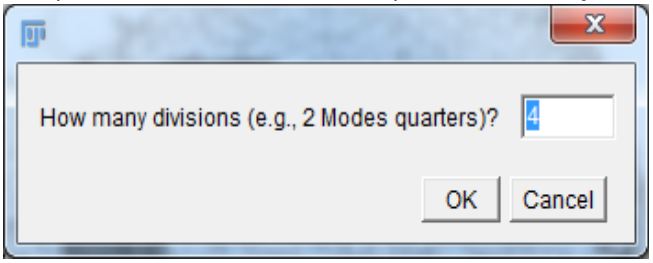

9. **Wait for Myosoft processing.**
You'll see the program rapidly highlighting cells from left to right, after at least 15 minutes. If possible, don't disturb Myosoft, such as clicking image processing window.

10. **Determine gate for each fiber type.**

After Myosoft has fully processed the images, prompts will appear to determine the intensity gates of the I, IIa and IIb reference images. DO NOT CLICK OK until you have determined the intensity gate value!!! Once you select okay, you will not be able to interact with the histogram or reference image. The user can mouse over the Mean Distribution histogram bins to identify the bin with the minimum value between the two maximum value bins. Mousing over a bin will display its intensity value below the histogram. Additionally, the intensity value can be confirmed by mousing over pixels in the reference image. The user can compare the intensities of pixels that are stained versus aren't stained to confirm the intensity value determined by the histogram. **Once the intensity gate is determined the value can be inputted after clicking OK.** After inputting the intensity gate value, select OK. More prompts will appear to determine the

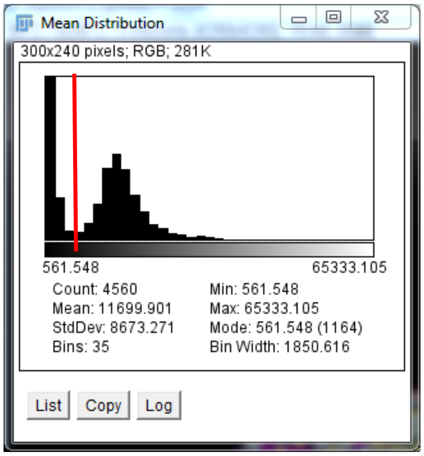

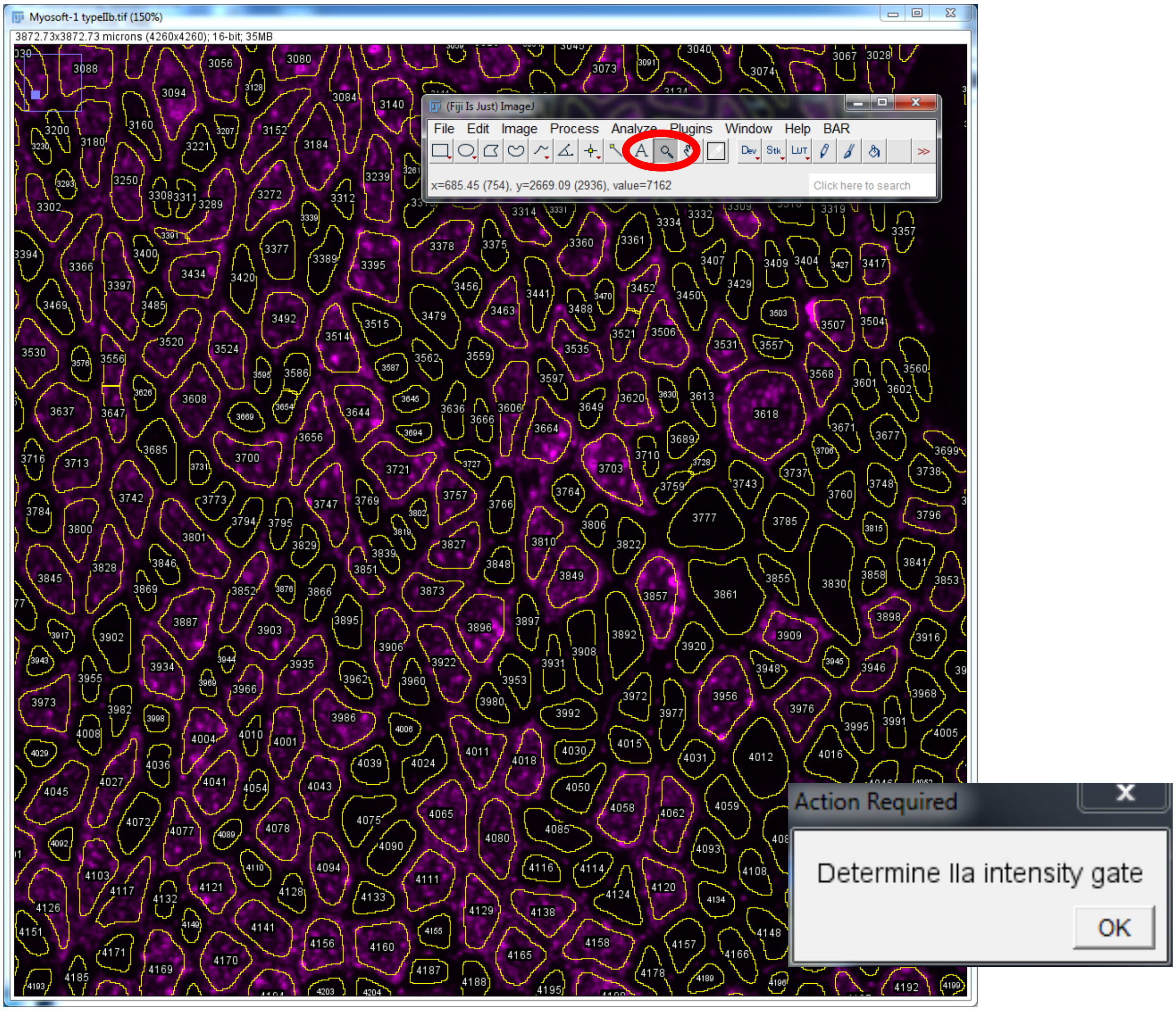

intensity gates the next two channels. 3 intensity gates will be determined corresponding to the Type I, Type IIa, and Type IIb fiber types.

11. **Analysis complete.**
After Myosoft analyzes the three channels for each fiber type and mixed fiber types, a pop-up box will display the message “Analysis Complete.” Analysis of ~10000 fibers (starting image ~6x6mm) takes 30 minutes on a computer with an i7 (3 GHz) processor and 16 GB RAM.

<u>Results</u>

1. Myosoft will generate individual spreadsheets (.csv format) with data from each channel in the original input image.
    a. The file from the membrane counterstain will be called “Fiber_Area_Results.”
    b. Each of the other files will be named with the fiber type(s) in the title

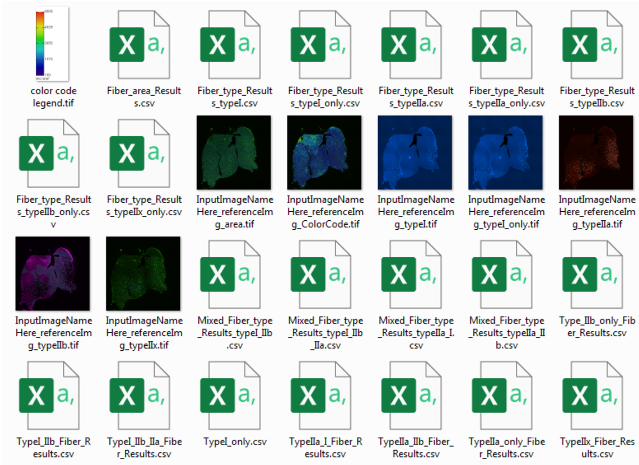

        i. “TypeIIa_only_Fiber_Results”
        ii. “TypeI_only_Fiber_Results”
        iii. “Type_IIb_only_Fiber_Results”
        iv. “TypeIIx_Fiber_Results”
        v. “TypeIIa_I_Fiber_Results”
        vi. “TypeIIa_IIb_Fiber_Results”
        vii. “TypeI_IIb_Fiber_Results”
        viii. “TypeI_IIb_IIa_Fiber_Results”

    c. In these files, each row represents a fiber identified by Myosoft, and all columns represent data for that fiber. Mean, StDev, Mode, Min and Max refer to the measured intensity from within the bounds of the fiber, while most of the other values are physical parameters (e.g. area, x/y position, min. feret).
2. Myosoft will generate a series of reference images. The reference images are single channel images from the original input image with “fiber boundaries” and numbers overlaid as a mask. The number of each object in a reference image corresponds to the same object number in the corresponding data spreadsheet. These images are provided so that users can visually inspect the objects from which measurements were taken by Myosoft.
3. Myosoft will generate ROI.zip files for each single fiber type and mixed fiber type. If the analyzed image is opened in FIJI, dragging the ROI.zip folder of any of the fiber types onto ImageJ will load the corresponding ROIs and will overlay the fiber boundaries of those fibers.
4. Myosoft will generate a color-coded version of your input image where different colors indicate different sized fibers. A colored scale indicator is also generated and saved in the location the user specifies for data (Step 6).

<u>About the Author</u>

Lucas Encarnacion-Rivera is currently an undergraduate student at Emory University. He may answer questions about Myosoft or help with troubleshooting if his schedule allows.
