# Supplementary figures and images for: Myosoft: An automated muscle histology analysis tool using machine learning algorithm utilizing FIJI/ImageJ software
Source: PLoS One. 2020 Mar 4;15(3):e0229041. doi: 10.1371/journal.pone.0229041 (PMC7055860; doi:10.1371/journal.pone.0229041)

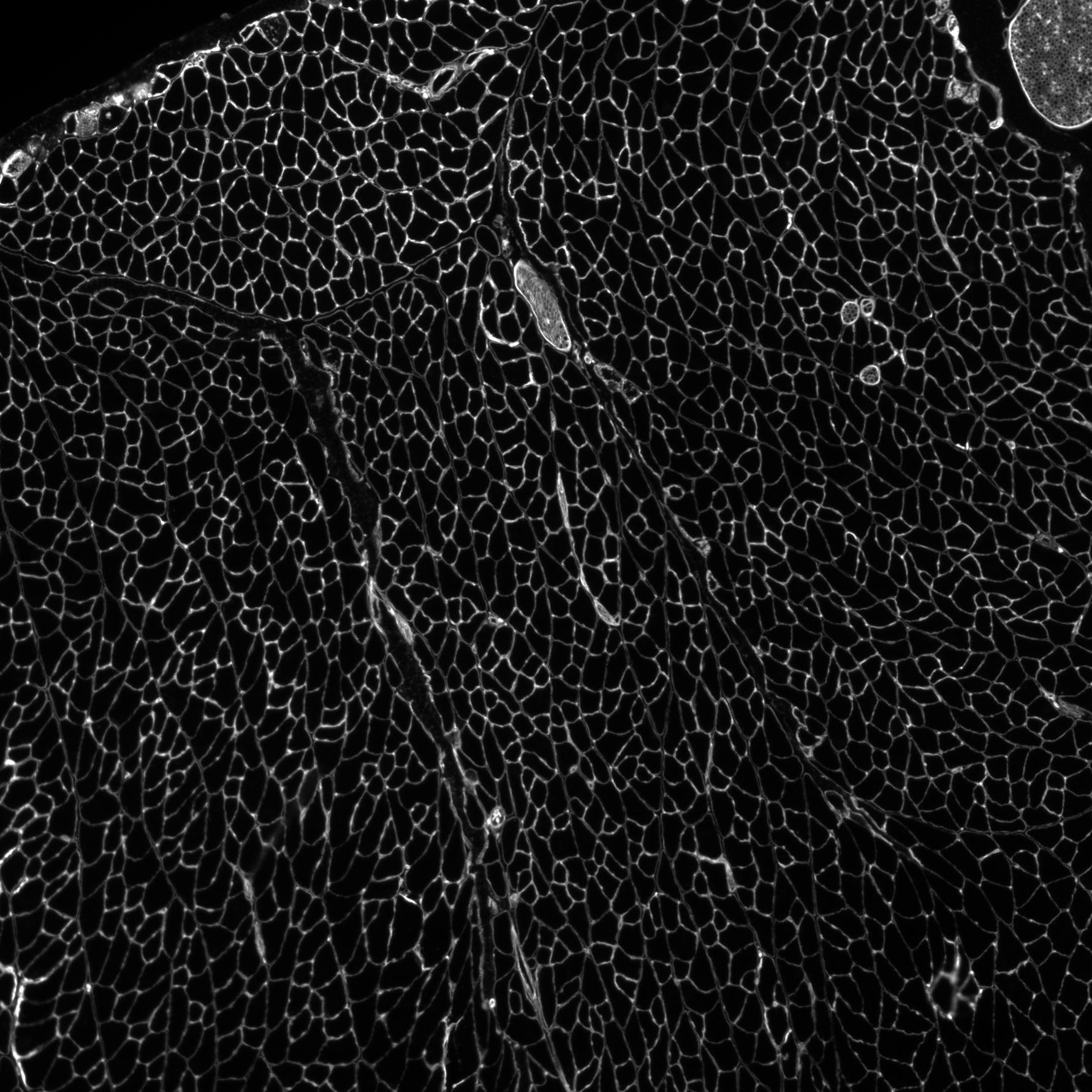

Supplement: S1 Fig — (TIF) [file pone.0229041.s004.tif]

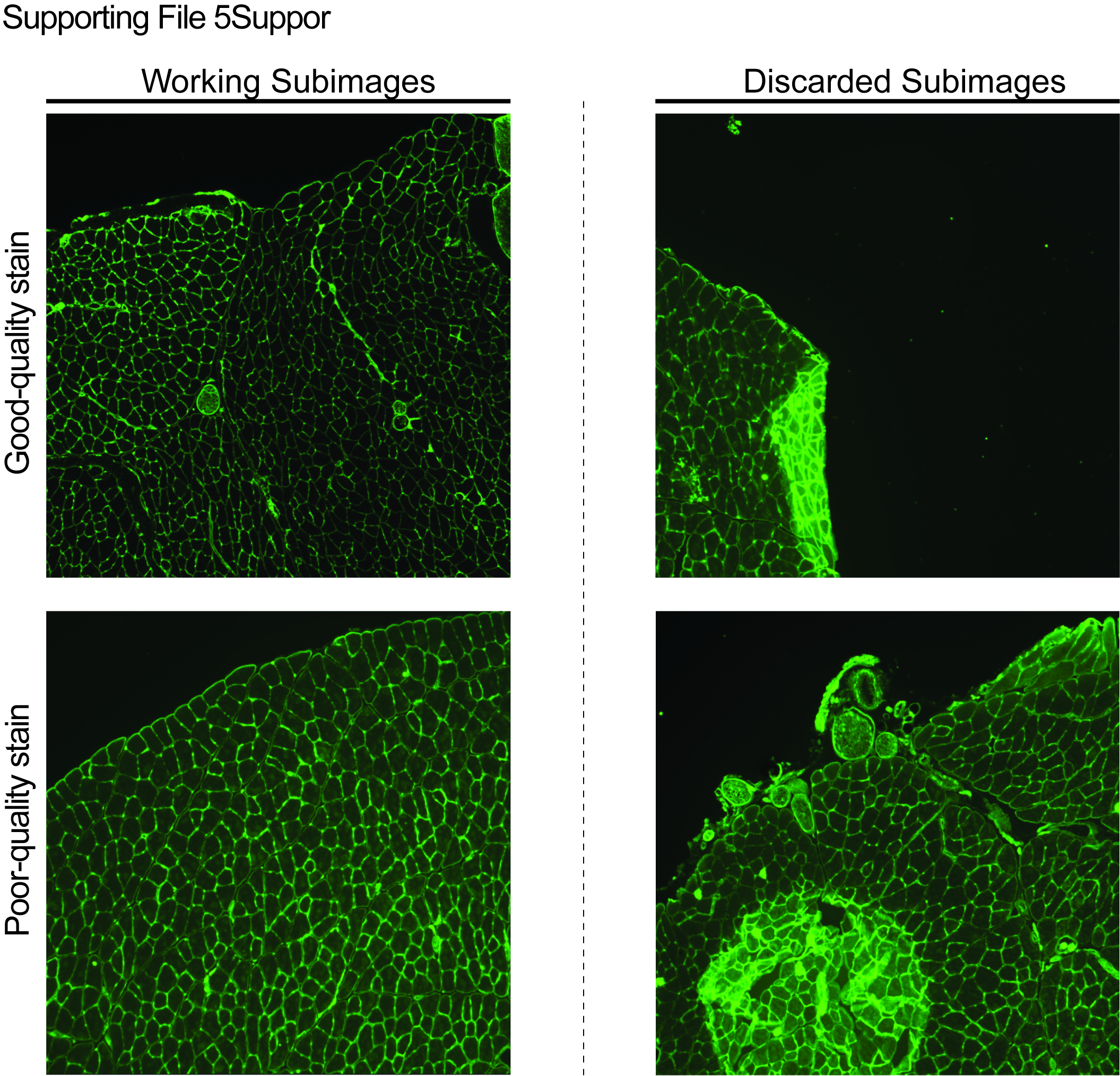

Supplement: S2 Fig — (TIF) [file pone.0229041.s005.tif]
