# Supplementary material for: Myosoft: An automated muscle histology analysis tool using machine learning algorithm utilizing FIJI/ImageJ software
Source: PLoS One. 2020 Mar 4;15(3):e0229041. doi: 10.1371/journal.pone.0229041 (PMC7055860; doi:10.1371/journal.pone.0229041)
Supplement: S1 Table — (DOCX) [file pone.0229041.s006.docx]

|  | No. of sections | Dimension of sections  (range, mm^2^) | No. of sub-images | Dimension of sub-images  (range, mm^2^) |
| --- | --- | --- | --- | --- |
| Figure 1 | 1 | 19.6 | 16 | 2.11 |
| Figure 2 | 1 | 19.6 | 0 | N/A |
| Figure 3 | 1 |  | 4 | 4 |
| Figure 4 | 3 | 8.2-12 | 6 (total) | 0.31 - 1.86 |
| Figure 5 | 16 | 8.4-17.8 | 22 (total) | 2.11 - 11.44 |
| Figure 6 | 8 | 8.8-15.3 | 128 (total) | 1.37 -2.11 |
| Figure 7 | 5 | 6.5-10.8 | 80 (total) | 1.37 |
| Total | 32 (unique) |  | 256 (unique) |  |

**Supporting file 6. Summary table of number of images for each figure**
